# The development of a prediction model based on deep learning for prognosis prediction of gastrointestinal stromal tumor: a SEER-based study

**DOI:** 10.1038/s41598-024-56701-2

**Published:** 2024-03-19

**Authors:** Junjie Zeng, Kai Li, Fengyu Cao, Yongbin Zheng

**Affiliations:** Department of Gastrointestinal Surgery, Renmin Hospital of Wuhan University, Wuhan, 430060 Hubei China

**Keywords:** Deep learning, Machine learning, Gastrointestinal stromal tumor, The surveillance, Epidemiology, And end results program (SEER), DeepSurv, Gastroenterology, Oncology, Risk factors

## Abstract

Accurately predicting the prognosis of Gastrointestinal stromal tumor (GIST) patients is an important task. The goal of this study was to create and assess models for GIST patients' survival patients using the Surveillance, Epidemiology, and End Results Program (SEER) database based on the three different deep learning models. Four thousand five hundred thirty-eight patients were enrolled in this study and divided into training and test cohorts with a 7:3 ratio; the training cohort was used to develop three different models, including Cox regression, RSF, and DeepSurv model. Test cohort was used to evaluate model performance using c-index, Brier scores, calibration, and the area under the curve (AUC). The net benefits at risk score stratification of GIST patients based on the optimal model was compared with the traditional AJCC staging system using decision curve analysis (DCA). The clinical usefulness of risk score stratification compared to AJCC tumor staging was further assessed using the Net Reclassification Index (NRI) and Integrated Discrimination Improvement (IDI). The DeepSurv model predicted cancer-specific survival (CSS) in GIST patients showed a higher c-index (0.825), lower Brier scores (0.142), and greater AUC of receiver operating characteristic (ROC) analysis (1-year ROC:0.898; 3-year:0.853, and 5-year ROC: 0.856). The calibration plots demonstrated good agreement between the DeepSurv model's forecast and actual results. The NRI values ( training cohort: 0.425 for 1-year, 0.329 for 3-year and 0.264 for 5-year CSS prediction; test cohort:0.552 for 1-year,0.309 for 3-year and 0.255 for 5-year CSS prediction) and IDI (training cohort: 0.130 for 1-year,0.141 for 5-year and 0.155 for 10-year CSS prediction; test cohort: 0.154 for 1-year,0.159 for 3-year and 0.159 for 5-year CSS prediction) indicated that the risk score stratification performed significantly better than the AJCC staging alone (P < 0.001). DCA demonstrated the risk score stratification as more clinically beneficial and discriminatory than AJCC staging. Finally, an interactive native web-based prediction tool was constructed for the survival prediction of GIST patients. This study established a high-performance prediction model for projecting GIST patients based on deep learning, which has advantages in predicting each person's prognosis and risk stratification.

## Introduction

With an annual prevalence of 11 to 19.6 per million people globally, gastrointestinal stromal tumors (GIST) are among the most prevalent mesenchymal malignancies in the digestive system^1^. They are usually found in the stomach or small intestine and rarely in other parts of the abdomen. Gastrointestinal mesenchymal tumors can develop at any age, with a median age of 60–65 years, and males and females are impacted at similar rates. GIST is a heterogeneous group of tumors, approximately 80% of which are mutually exclusive activating KIT or PDGFRA mutations, but other rare subtypes also exist^2^. Surgery is the treatment for Gist, but the higher recurrence rate remains troubling^3^. It is usually not straightforward to undergo surgery for advanced cases, and GIST is resistant to standard cytotoxic treatments used for other sarcomas^4–7^. Although targeted drug therapy can contribute to patients' prognosis, some risks are associated with continuous therapy^8,9^. Therefore, identifying patients with poor prognoses is particularly important, and clinically high-risk patients deserve more attention.

The TNM staging system is widely used in clinical practice to provide prognostic predictions based on tumor size, lymph node involvement, and distant metastasis. However, the TNM staging system's clinical value is diminished because lymph node involvement is rare in GIST^10^, and mitotic index, tumor location, and other tumor dimensions are not considered^11^. The homogeneity of prediction algorithms may hamper the effectiveness of existing models for determining the prognosis of GIST patients. These models rely on nomograms based on Cox risk regression algorithms^12–15^.

Deep learning networks can uncover complex linear and nonlinear relationships between predictive clinical characteristics and an individual's risk of death^16^. These neural networks have even demonstrated the ability to give specific recommendations based on assessed applied risk^17^. In addition, Katzman et al. created the Deep Learning Survival Neural Network (DeepSurv)^18^, a new deep learning survival analysis method that integrates Cox proportional risk. The authors demonstrated that DeepSurv performs as well as, if not better than, existing survival models and can be used to prescribe treatments for better survival outcomes. There have been a number of studies using deep learning techniques for survival prognosis of tumor patients, but the use of Deepsurv for prognostic analysis of gastrointestinal mesenchymal tumors has not yet been reported^19–22^.

In this study, we aim to use data from the Surveillance, Epidemiology, and End Results Program (SEER) database and propose a prognostic model for GIST based on deep learning algorithms. Additionally, we work to give doctors and patients prognostic tools to evaluate each GIST patient's prognosis on an individual basis.

## Materials and methods

### Study population

**Figure 1:**
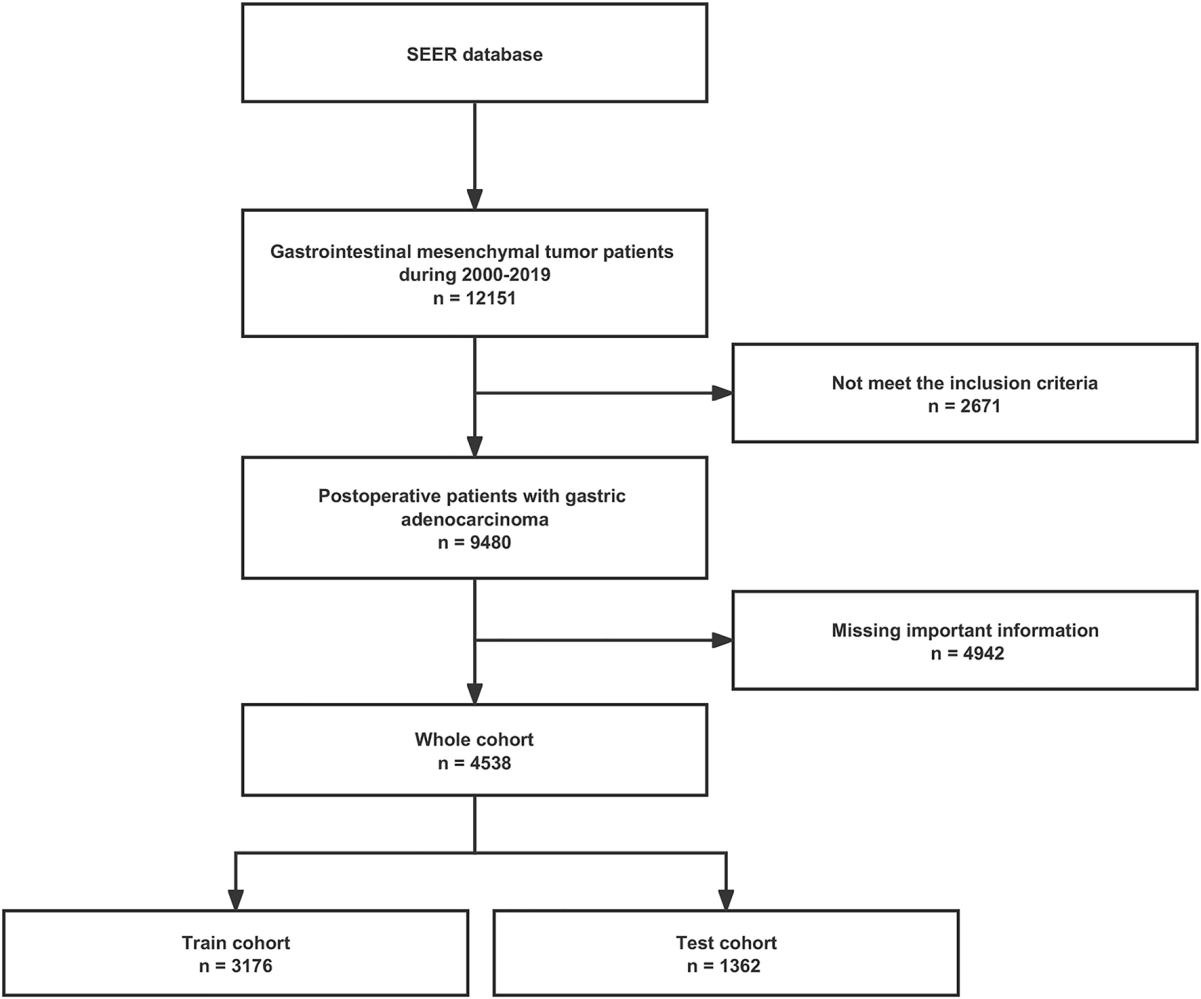
The flowchart of data filtering.

The SEER public database is representative of the US population, and patient data were obtained from multi-center population data such as rural and urban. Gastrointestinal mesenchymal tumor cases and their details were retrieved between 2000 and 2019 using SEER*Stat version 8.4.1 software. The International Classification of Diseases code version 3 (ICD-O-8936), histological histological code 8936/3 and topographical codes C15.0, C15.1, C15.2, C15.3, C15.4, C15.5, C15.9, C16.0, C16.1, c16.2, c16.3, c16.4, c16.5, c16.6, c16.9, c17.0, c17.1, c17.2, c17.3, c17.9, c18.0, 18.1, c18.2, c18.3, c18.4, c18.5, c18.6, c18.7, c18.9, c19.9, c20.9, C21.0, C21.1, and C21.2 for case information. Malignant tumor behavioral coding was employed for analysis. Exclusion standards included (1) cases without pathologic confirmation, (2) patients younger than 20 years of age, (3) gastrointestinal mesenchymal tumors that were not the first primary tumor, and (4) missing relevant clinical or pathological information, such as surgical information, information on the primary site of the tumor, and information on the AJCC stage information level of follow-up. The study's main concern was the time between the initial diagnosis and death from the gastrointestinal mesenchymal tumor. This is known as cancer-specific survival (CSS). The data screening process is exhibited in Fig. 1.

### Variables

The study incorporated the following variables: age, gender, race, marital status, primary tumor site, tumor size, mitosis, SEER stage, AJCC stage (based on official SEER database entries), and tumor pathology grading. Treatment-related information, including surgery, chemotherapy, and radiation therapy, was also incorporated. According to the official dictionary of the SEER database, the Mitosis field "High" is defined as Over five mitoses per 5 square mm; "Low" is defined as five or fewer mitoses.

### The development of models

**Figure 2:**
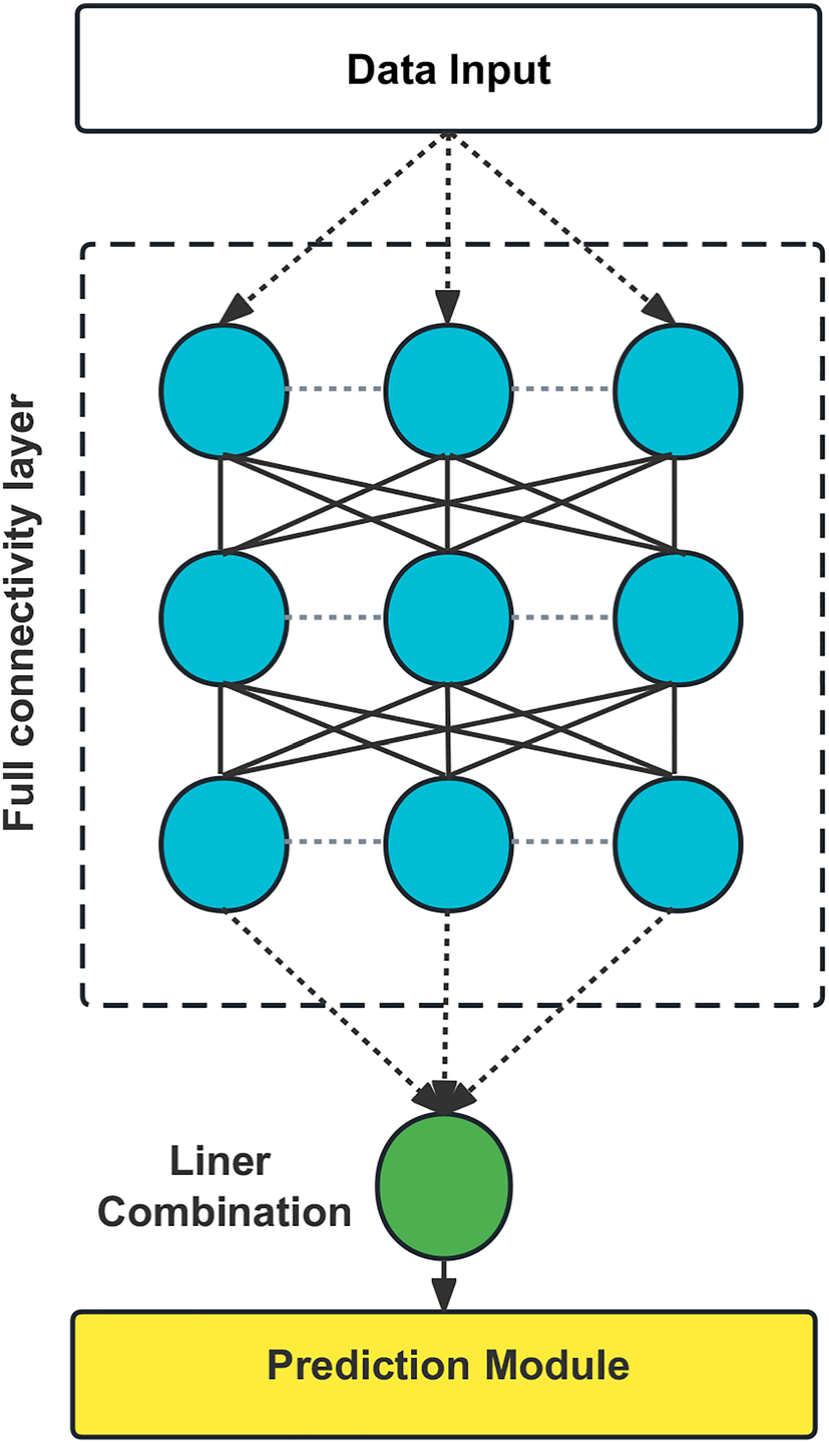
Diagram of the deep learning procedure.

The cases were randomly divided into training and test sets in a ratio of 7:3. Three different prediction models were constructed in this study, namely, the Cox risk regression model based on the linear prediction model^23^, Random Survival Forest (RSF) model based on the machine learning algorithm and DeepSurv model based on a deep learning algorithm^24–26^. The three prediction models are based on the algorithm's characteristics to select suitable variables for the best prediction performance. Cox regression model variable selection is based on single-factor and multi-factor regression analysis. In contrast, the RSF model uses lattice search in combination with K-cross validation to select the best combination of variables. The DeepSurv model is based on a neural network algorithm, where all the variables can be included in the training directly without needing selection. Meanwhile, grid search optimizes RSF and DeepSurv models with hyperparameters. Model training and hyperparameter tuning are done on the training set (Fig. 2).

### The evaluation and interpretation of models

The model's predictive performance was evaluated on the test set. The metrics used included the consistency index (c-index), a subject work characterization curve (ROC curve), a Brier score over time, and a calibration curve. A well-calibrated model should have a Brier score of less than 0.25. The study ranked the importance of the features of the models with superior mapping predictive performance to expect an interpretation of the models.

### The DeepSurv risk stratification of patients

DeepSurv risk stratification is based on a risk score, which is calculated by a trained model based on the number of endpoint events and expected events to quantify the survival risk of patients. Risk scores were stratified using X-tile software to screen for optimal cutoff values. DeepSurv risk stratification was tested by Kaplan–Meier survival analysis and log-rank test.

The clinical advantages and usefulness of the DeepSurv risk stratification in comparison to AJCC tumor staging alone were assessed using the net reclassification index (NRI), integrated discrimination improvement (IDI), and decision curve analysis (DCA). NRI and IDI are two substitutes for AUC that may be used to gauge how well a new model predicts risks and how beneficial it is^27,28^. DCA is a technique for estimating net benefits at various threshold probabilities and assessing the clinical value of alternative models^29,30^. The curves for the treat-none system (representing no clinical benefit) and the treat-all-patients scheme (showing the highest clinical expenses) were presented as two references.

### The individual prediction

The study constructed an interactive local web tool to provide individualized survival prediction. The web tool consists of two primary interfaces: (1) the patient information input interface and (2) the prediction result display interface. After completing the model-related variable information input in the information input interface according to the instruction, clicking the prediction button will display the patient's expected survival curve and 1-year, 3-year, and 5-year survival rates in the result display interface. The prediction tool also provides the function of displaying data from multiple cases simultaneously.

### Statistical analysis

Differences in demographic and clinical data between the training and validation sets were evaluated using the Wilcoxon test for continuous variables and the χ^2^ test or Fisher's exact test for categorical variables. Statistical significance was defined as a two-tailed p-value less than 0.05. Python (version 3.7) was used to derive the models. The Cox, RSF, and DeepSurv models were based on the pysurvival modules (version 0.17.2, Sebastian P). Primary data analysis was performed by R (version 4.2.3) and SPSS—data visualization based on GraphPad Prism 9. The interactive prediction tool relied on Streamlit (https://streamlit.io/) for its construction.

### Ethics approval

Because the SEER database is a publicly available database of de-identified patient data, no ethics committee review was required for its use in this project.

## Results

### The characteristics of patients

In this study, 4538 patients in total were examined. Table 1 demonstrates the primary baseline information of the patients and their grouping between the training and test sets. The majority of patients were white (3042, 67.03%). In terms of gender, males were approximately equally distributed to females. Regarding the tumor primary site, the highest percentage of patients had a prior site in the stomach (2932, 64.60%), followed by patients with a gastric primary site in the small intestine (1397, 30.78%). The distribution of patients in the training and test sets was approximately the same and did not demonstrate significant differences (p > 0.05).

**Table 1:** The information for Gastrointestinal stromal tumor patients in the training cohort and the test cohort.

Characteristic	Train cohort	Test cohort	*p*-value
	n = 3176	n = 1362	
Age			0.8523
20–49 years	544(17.13%)	224(16.45%)	
50–69 years	1652(52.02%)	713(52.35%)	
70 + years	980(30.86%)	425(31.20%)	
Gender			0.7507
Female	1563(49.21%)	678(49.78%)	
Male	1613(50.79%)	684(50.22%)	
Race			0.4087
Asian or Pacific Islander	476(14.99%)	200(14.68%)	
Black	558(17.57%)	262(19.24%)	
White	2142(67.44%)	900(66.08%)	
Marital			0.2586
Married	1876(59.07%)	795(58.37%)	
Single	560(17.63%)	222(16.30%)	
Other/Unknown	740(23.30%)	345(25.33%)	
Primary site			0.3339
Colon	43(1.35%)	30(2.20%)	
Esophagus	14(0.44%)	7(0.51%)	
Rectum	90(2.83%)	25(1.84%)	
Small Intestine	999(31.45%)	398(29.22%)	
Stomach	2030(63.92%)	902(66.23%)	
Grade			0.736
Well differentiated	669(21.06%)	287(21.07%)	
Moderately differentiated	406(12.78%)	177(13.00%)	
Poorly differentiated	122(3.84%)	49(3.60%)	
Undifferentiated	190(5.98%)	68(4.99%)	
Unknown	1789(56.33%)	781(57.34%)	
SEER stage			0.9536
Distant	574(18.07%)	241(17.69%)	
Localized	2243(70.63%)	967(71.00%)	
Regional	359(11.30%)	154(11.31%)	
Mitosis			0.5025
High	166(5.23%)	75(5.51%)	
Low	446(14.04%)	174(12.78%)	
Unknown	2564(80.73%)	1113(81.72%)	
Tumor size			0.5697
< 5 cm	1321(41.59%)	586(43.02%)	
5–9.9 cm	1048(33.00%)	429(31.50%)	
10 + cm	807(25.41%)	347(25.48%)	
AJCC stage			0.959
I	1525(48.02%)	662(48.60%)	
II	526(16.56%)	217(15.93%)	
III	552(17.38%)	238(17.47%)	
IV	573(18.04%)	245(17.99%)	
Radiation			0.5625
No	3161(99.53%)	1353(99.34%)	
Yes	15(0.47%)	9(0.66%)	
Chemotherapy			0.573
No	1695(53.37%)	740(54.33%)	
Yes	1481(46.63%)	622(45.67%)	
Surgery			0.1378
No	298(9.38%)	148(10.87%)	
Yes	2878(90.62%)	1214(89.13%)	

### The development of models

Cox model: according to univariate Cox regression analysis, age, gender, tumor primary site, tumor pathology grading, Seer grading, tumor size, AJCC staging, chemotherapy, radiation therapy, and surgery were significant variables. Age, tumor size, AJCC stage, chemotherapy, and surgery were considered significant variables in multifactorial regression analysis. Detailed information is displayed in Tables S1 and S2. RSF model: based on grid search and K-fold cross-validation, the optimal combination of variables for the model was determined to be tumor size, AJCC stage, SEER stage, pathology grading, surgery information, age, chemotherapy information, gender, tumor primary site, mitotic rate, marital status, race, and radiotherapy information. Variable selection details are shown in Table S3.

DeepSurv model: the model neural network structure was finalized after grid search. The model structure is simplified and displayed in Fig. 2, while the model details are uploaded to Git Hub (https://github.com/DrZJJ/GIST.git).

### The evaluation and interpretation of the models

**Figure 3:**
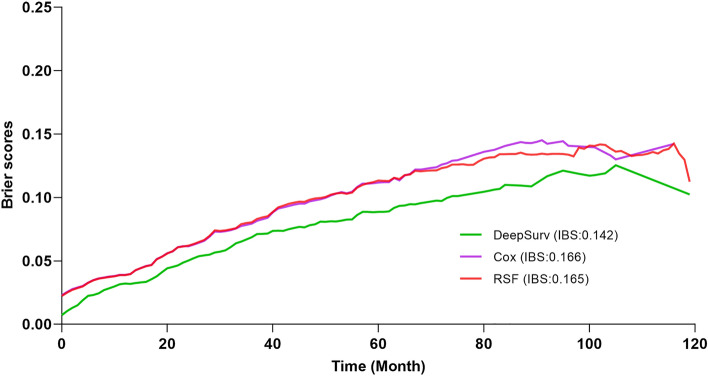
Prediction error curve. A useful model will have a Brier score less than 0.25 as a standard.

In the test set, the model's effectiveness is assessed. As can be seen in Fig. 3, the Brier scores of each model are below 0.25, demonstrating good accuracy. The DeepSurv model has the smallest Brier score at all time points, demonstrating an advantage. Regarding the C-index, the DeepSurv model is highest at 0.825 (Table 2).

**Table 2:** The models' performance in the Test cohort.

Model	Brier score			C-index
	1-year	3-year	5-year	
Cox model	0.039	0.079	0.112	0.761
RSF model	0.039	0.08	0.113	0.789
DeepSurv model	0.032	0.069	0.089	0.825

**Figure 4:**
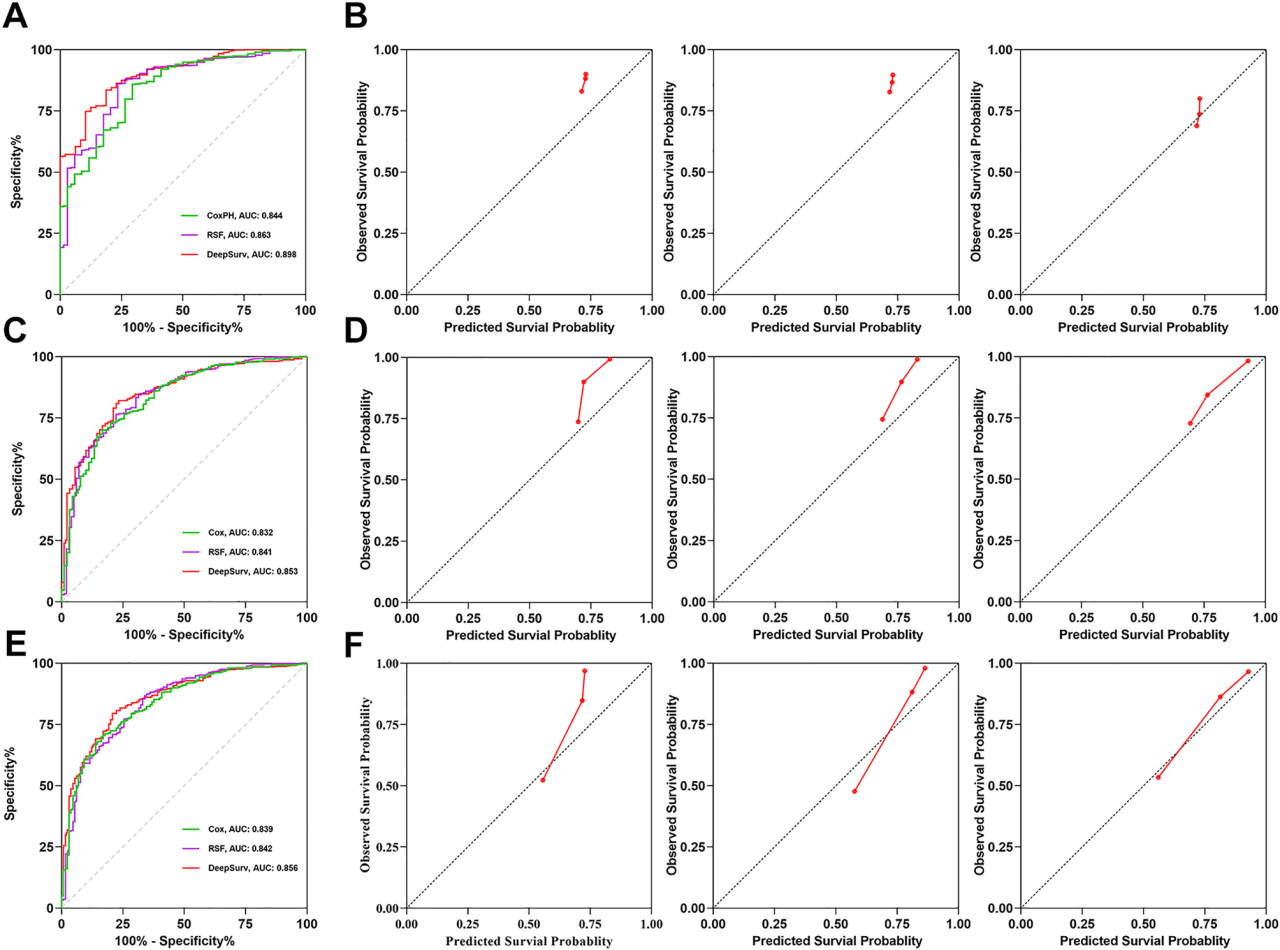
The receiver operating curves (ROC) and calibration curves for 1-, 3-, 5-year survival predictions. ROC curves for (**A**) 1-, (**C**) 3-, (**E**) 5-survival predictions. Calibration curves for (**B**) 1-, (**D**) 3-, (**F**) 5-year survival predictions. In (**B**), (**D**), and (**F**), each set of images is arranged in the order of Cox model, RSF model, and DeepSurv model.

The ROC and calibration curves for each model at 1, 3, and 5 years are shown in Fig. 4. The area under the curve for the DeepSurv model is 0.898, 0.8528, and 0.8564, respectively, higher than that of the Cox and RSF models. The calibration curves of each model are shown in Fig. 4, and the DeepSurv model calibration curve is closer to the diagonal line, offering an advantage.

### The DeepSurv risk stratification of patients

**Figure 5:**
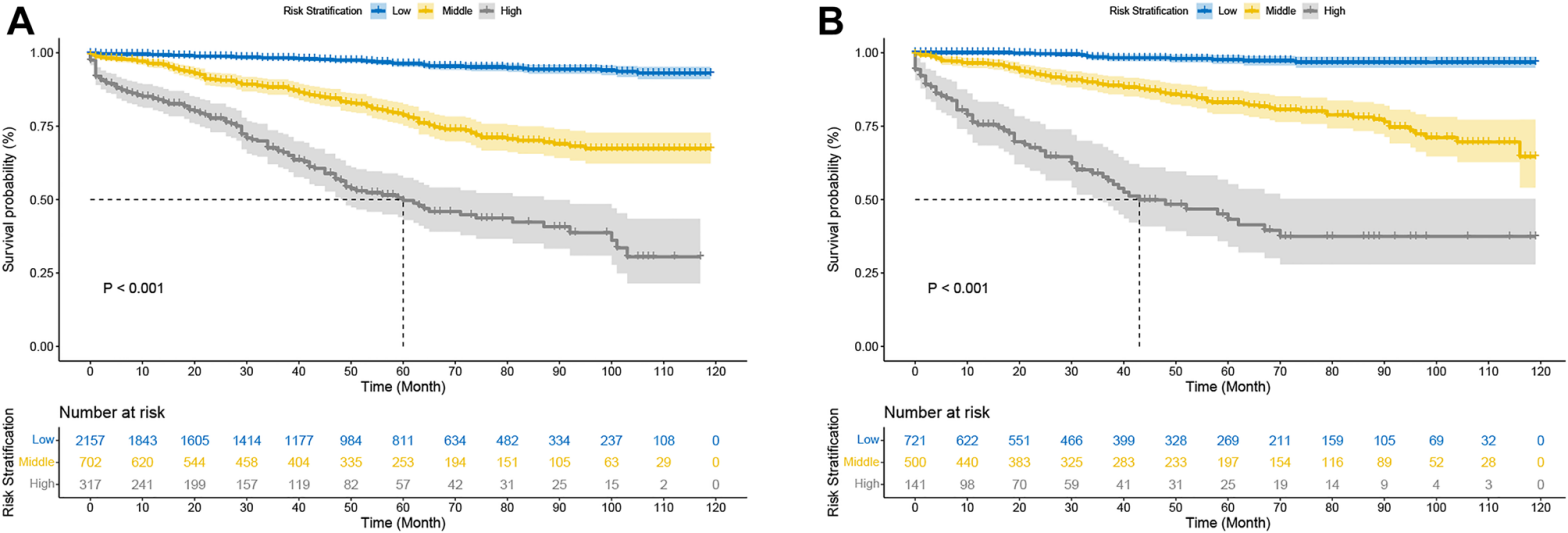
Kaplan–Meier curves of cancer-specific survival for new risk classification and the AJCC tumor staging (**A**) The AJCC stage in the test cohort; (**B**) The deepsurv risk stratification in the test cohort.

Stratification of patients is essential to guide clinical management. The optimal thresholds for DeepSurv risk stratification were determined based on the X-tile software. X-tile is a bioinformatics tool for biomarker assessment and outcome-based cut-point optimization. With the help of the X-tile, patients were categorized into the high-risk group (> 46), medium-risk group (8–46), and low-risk group (8 <). More information about the X-tile is described in additional material. The results of the Kaplan–Meier survival analysis and log-rank test for high-risk, intermediate, and low-risk groups are displayed in Fig. 5. The Kaplan‐Meier CSS curves showed significant discrimination among the three risk groups in the training and testing cohorts.

The C-index, NRI, and IDI changes were used to compare the accuracy between the risk stratification and the AJCC staging alone. While using the risk stratification in the training cohort, the C-index was 0.840, the NRI for the 1-, 3- and 5-year CSS were 0.425 (95% CI = 0.006‐0.564),0.329 (95% CI 0.214–0.449) and 0.264 (95% CI 0.167–0.365), and the IDI values for 1-, 3- and 5-year CSS were 0.130 (95% CI 0.116–0.146, P < 0.001),0.141 (95% CI 0.119–0.165, P < 0.001) and 0.155 (95% CI 0.125–0.191, P < 0.001) (Table 3). These findings, which showed that the DeepSurv risk stratification predicted prognosis more effectively than the AJCC staging, were verified in the testing group (Table 3).

**Table 3:** C‐index, NRI, and IDI of the DeepSurv risk stratification and AJCC stage in survival prediction for GIST patients.

Index	Training cohort		P value	Testing cohort		P value
	Estimate	95% CI		Estimate	95% CI	
NRI (vs. the AJCC tumor staging)						
For 1‐year CSS	0.425	0.006–0.564		0.552	0.042–0.741	
For 3‐year CSS	0.329	0.214–0.449		0.309	0.120–0.453	
For 5‐year CSS	0.263	0.167–0.365		0.255	0.127–0.430	
IDI (vs. the AJCC tumor staging)						
For 1‐year CSS	0.130	0.116–0.146	** < 0.001**	0.154	0.125–0.187	** < 0.001**
For 3‐year CSS	0.141	0.119–0.165	** < 0.001**	0.159	0.113–0.199	** < 0.001**
For 5‐year CSS	0.155	0.125–0.191	** < 0.001**	0.159	0.110–0.204	** < 0.001**
C‐index						
The risk stratification	0.840			0.811		
The AJCC tumor staging	0.774			0.784		

Significant values are in bold.

**Figure 6:**
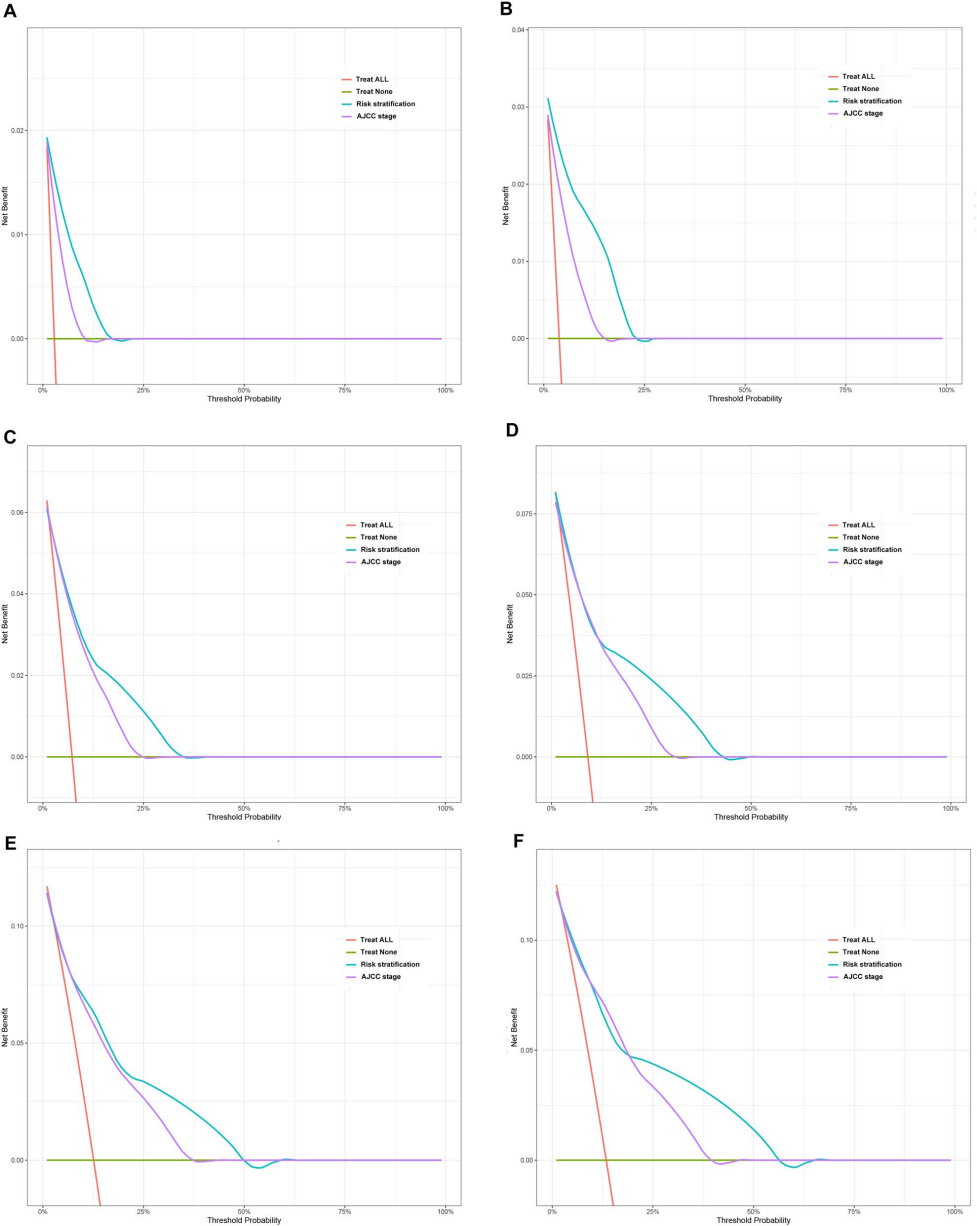
Decision curve analysis of the DeepSurv risk stratification and AJCC tumor staging for the survival prediction of GIST patients. (**A**,**C**,**E**) 1‐year, 3‐year and 5‐year survival benefit in the train cohort. (**B**,**D**,**F**) 1‐year, 3‐year and 5‐year survival benefit in the test cohort.

The clinical benefits of the risk stratification were compared with those of the AJCC stage. DCA curves showed that the risk stratification could better predict the 1-, 3- and 5-year CSS, as it added more net benefits compared with the AJCC stage for almost all threshold probabilities in both the training and validation cohorts, and with both the treat-all-patients scheme and the treat-none scheme (Fig. 6).

**Figure 7:**
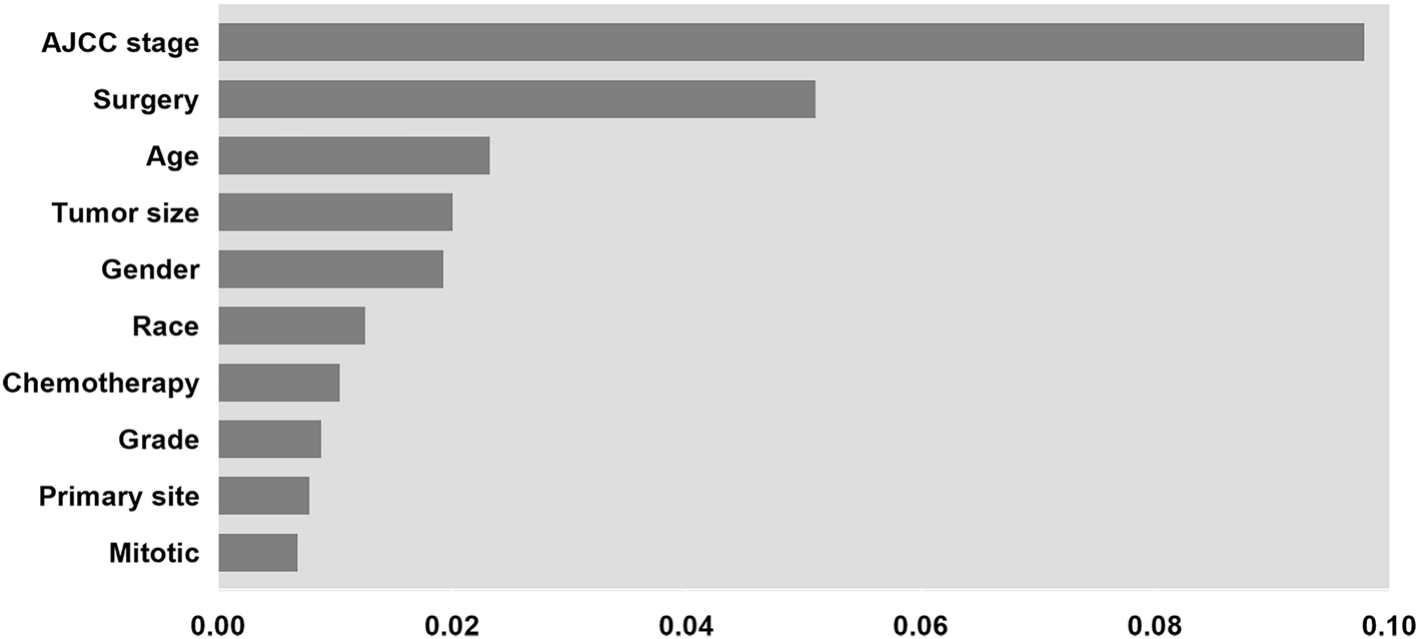
Feature importance for DeepSurv model, only the top 10 variables in importance are shown in the Figure.

In addition, the DeepSurv model was interpreted visually. The importance of the variables in the model was mapped, and in Fig. 7, the variables located in the top 10 are displayed in descending order.

### The individual postoperative prognostic prediction

**Figure 8:**
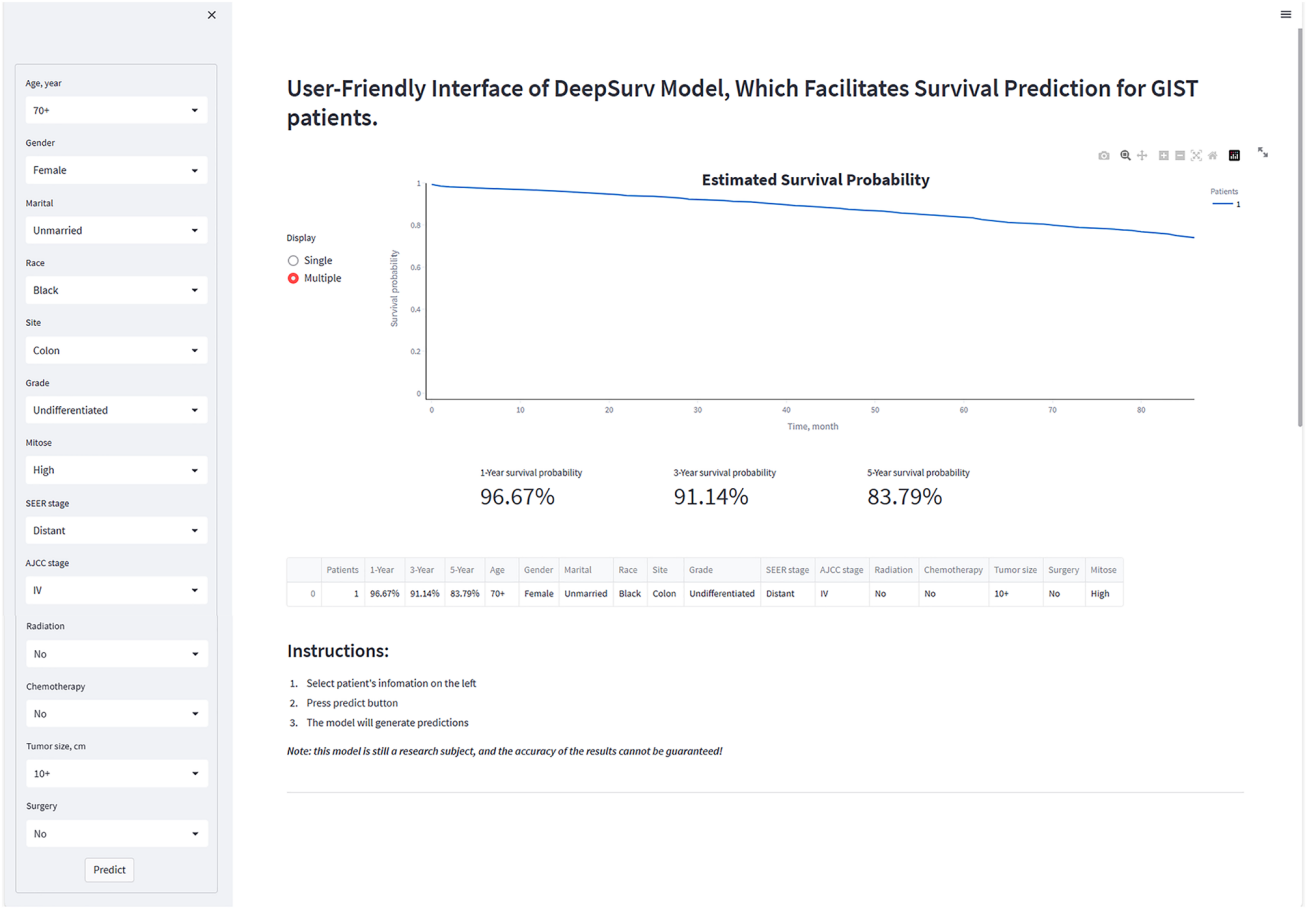
The manual interactive interface based on Deepsurv model for predicting the survival probabilities of GIST patients.

The study developed a manual interactive interface based on a trained Deepsurv model for predicting the survival probabilities of GIST patients (Fig. 8). The analysis results are visualized in a graphical view as a survival curve that shows the survival probability of the patient's input data over time and highlights the patient's survival rates at 1, 3, and 5 years postoperatively at the bottom of the graph. In addition, it is possible to fit survival curves for different patients to the same chart to compare patients easily. (Github: https://github.com/DrZJJ/GIST.git).

## Discussion

This study created a deep learning-based prognostic model for GIST utilizing the SEER database. When predicting 1-, 3-, and 5-year CSS in GIST patients, the DeepSurv model outperformed Cox regression and RSF model in calibration and discrimination. By ranking the importance of model features, AJCC stage, age, surgery, and tumor size were essential variables affecting the prediction. In addition, risk stratification and individual prognosis prediction based on DeepSurv models showed potential in clinical practice.

Previously, survival and speculation about GIST patients were mainly performed based on linear models. However, these models incorporate fewer risk factors significantly associated with survival or recurrence. Deep learning techniques have been widely used as a new tool to support clinical decision-making^16,31,32^. DeepSurv models, as an application of deep learning techniques, can analyze data with a more significant number of variables and integrate nonlinear functions associated with outcomes^33^. In addition, the DeepSurv model has a powerful representation learning capability to learn from unexplained clinical data automatically^34^. Compared with traditional regression analysis and random survival forests, DeepSurv algorithms build models with better performance, especially when dealing with high-dimensional and complex data, always showing impressive performance.

The feature importance ranking compares and presents the attributes that exhibit significance for model training^35^. Existing studies have found that GIST is more prevalent among Asian/Pacific Islanders or Blacks, with a more stable male-to-female ratio. GIST is diagnosed at any age, and there are differences in most molecular subtypes of GIST depending on the age group^36,37^. Although these variables show high levels of characterization importance, they are more valuable at the epidemiological level, and their role in patient prognosis still needs further investigation. Surgery and targeted therapy, as common clinical treatment modalities, effectively improve the prognosis of GIST patients^38–40^. Mitotic counting is also an essential factor in predicting GIST^41^. However, mitotic identification is more likely to be subjective and subject to error depending on how it is used^3,42^. The site of tumor origin is also an essential piece of information in the clinical management of patients with GIST; the prognosis of GIST in the colorectum is slightly worse than in the stomach, and when GIST is present in the abdominal cavity outside the gastrointestinal tract, then it is considered to have a high likelihood of an unfavorable outcome^43,44^.

DeepSurv risk stratification allows for assessing patient prognosis by integrating clinical information about the patient. AJCC staging is a primary tumor staging method that allows for a cursory assessment of patient prognosis. The differentiation of survival prediction for stage I, II, and III patients in this study was also less satisfactory. Compared with the well-established AJCC staging system, the DeeoSurv risk stratification in this study incorporates more variables. Also, it applies more novel deep learning algorithms in the algorithm, showing satisfactory results. DeepSurv risk stratification also provides a more flexible prediction method than column-line diagrams, widely used in oncology^45^. When using DeepSurv risk stratification, physicians can assess the survival cycle of patients to a certain extent and focus more on patients in high-risk groups to expect a better prognosis^46^. Of course, the construction of DeepSurv risk stratification is based on only 4538 patients included in the study, and its advantages and disadvantages over other scoring systems and its clinical utility still need to be evaluated on a broader population.

We should recognize some limitations of this study. First, the study was retrospective, and a potential selectivity bias may exist. Second, the accuracy and generalizability of the model still need to be tested using a substantial quantity of external patient data because both the training and test set data originated from the same database. Thirdly, the SEER database lacks certain crucial factors including chemotherapy drug type, chemotherapy regimen, patient adherence to chemotherapy, cancer cell margin status, and preoperative or intraoperative cancer rupture. Furthermore, more comprehensive tumor pathology and immunohistochemistry information (e.g., specific mutation details and mitotic rate) was not accessible. It is regrettable that these highly sought-after clinical care details are incomplete. The improved database information will significantly improve the model's prediction and risk differentiation ability; due to the lack of the above information, the model still needs to be further enhanced, which will be the focus of our future in-depth research.

## Conclusions

We created high-performance prediction models for the prognosis of GIST patients using deep learning techniques. In addition, we stratified the GIST population and comprehensively predicted individual prognosis using the DeepSurv model. We also provide an easy-to-use predictive tool for physicians and patients and promote personalized medicine. Our research supports deep learning algorithms and shows promise for future clinical practice.

### Supplementary Information

Supplementary Tables.

## Data Availability

The data used in this study can be retrieved and downloaded from the SEER database (https://seer.cancer.gov/).
