# Supplementary material for: The development of a prediction model based on deep learning for prognosis prediction of gastrointestinal stromal tumor: a SEER-based study
Source: Sci Rep. 2024 Mar 19;14:6609. doi: 10.1038/s41598-024-56701-2 (PMC10951333; doi:10.1038/s41598-024-56701-2)
Supplement: Supplementary file 1 — Supplementary Tables. [file 41598_2024_56701_MOESM1_ESM.docx]

**The Development of a Prediction Model Based on Deep Learning for prognosis prediction of** **Gastrointestinal stromal tumor: A SEER-Based Study**

Junjie Zeng, MD^1#^, Kai Li, MD^1#^, Fengyu Cao, MD^1^, Yongbin Zheng, MD, PhD^1^*

^1^Department of Gastrointestinal Surgery, Renmin Hospital of Wuhan University, Wuhan, Hubei, China.

^#^Junjie Zeng and Kai Li contributed equally to this work.

*Corresponding authors: Yongbin Zheng, MD, PhD, Department of Gastrointestinal Surgery, Renmin Hospital of Wuhan University, Wuhan, Hubei, 430060, China. Email: [yongbinzheng@whu.edu.cn](mailto:yongbinzheng@whu.edu.cn)

**Table S1** The results of the univariable Cox regression analysis

| Characteristics | HR | Lower.95 | Uper.95 | P value |
| --- | --- | --- | --- | --- |
| Age | 1.71 | 1.44 | 2.02 | <0.001 |
| Gender | 1.45 | 1.16 | 1.81 | <0.001 |
| Site | 0.85 | 0.75 | 0.98 | 0.02 |
| Grade | 1.20 | 1.12 | 1.28 | <0.001 |
| SEERstage | 0.34 | 0.28 | 0.41 | <0.001 |
| Tumor size | 2.48 | 2.14 | 2.88 | <0.001 |
| AJCCstage | 2.50 | 2.25 | 2.78 | <0.001 |
| Radiation | 4.47 | 1.85 | 10.81 | <0.001 |
| Chemotherapy | 1.84 | 1.47 | 2.30 | <0.001 |
| Surgery | 0.18 | 0.14 | 0.22 | <0.001 |

**Table S2** The results of the multivariable Cox regression analysis

| Characteristics | HR | Lower.95 | Uper.95 | P value |
| --- | --- | --- | --- | --- |
| Age | 1.73 | 1.46 | 2.06 | <0.001 |
| Gender | 1.13 | 0.90 | 1.41 | 0.28 |
| Site | 0.97 | 0.84 | 1.12 | 0.68 |
| Grade | 1.01 | 0.94 | 1.10 | 0.72 |
| SEERstage | 1.03 | 0.87 | 1.23 | 0.70 |
| Tumor size | 1.49 | 1.26 | 1.76 | <0.001 |
| AJCCstage | 2.17 | 1.88 | 2.51 | <0.001 |
| Radiation | 1.53 | 0.63 | 3.73 | 0.35 |
| Chemotherapy | 0.59 | 0.46 | 0.76 | <0.001 |
| Surgery | 0.41 | 0.31 | 0.54 | <0.001 |

**Table S3** RSF model variable selection results

| **K** | **Mean fit time** | **Mean score time** | **Mean test score** |
| --- | --- | --- | --- |
| 0 | 2.404998 | 0.033955 | 0.771921 |
| 1 | 2.45374 | 0.035065 | 0.767657 |
| 2 | 2.511405 | 0.036513 | 0.769165 |
| 3 | 2.67131 | 0.038959 | 0.767021 |
| 4 | 2.662275 | 0.039861 | 0.783298 |
| 5 | 2.660063 | 0.040228 | 0.804403 |
| 6 | 2.673461 | 0.040464 | 0.80661 |
| 7 | 2.672094 | 0.041908 | 0.810642 |
| 8 | 2.742799 | 0.040454 | 0.809862 |
| 9 | 2.931053 | 0.042483 | 0.811415 |
| 10 | 2.826889 | 0.039882 | 0.810813 |
| 11 | 2.807632 | 0.045806 | 0.812028 |
| 12 | 2.78077 | 0.042223 | 0.813376 |

The RSF model searches for the best combination of constituent model variables based on grid search and K-fold cross-validation. The best results were obtained for the 13th time when the composition of the variables was Age, Gender, Race, Marital, Site, Grade, SEER stage, Mitosis, Tumor size, AJCC stage, Radiation, Chemotherapy, and Surgery.


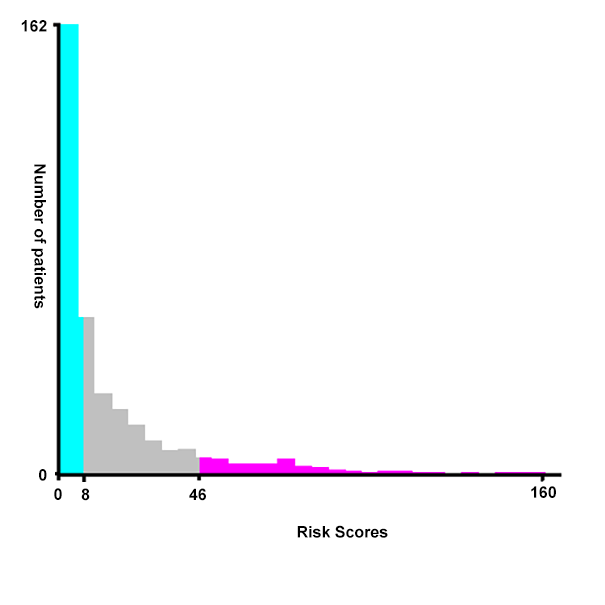


**Figure S1** The basis for grouping DeepSurv Risk Stratification (Cut-off point selected using X-tile).
